# Supplementary material for: The 12-Membered TNFR1 Peptide, as Well as the 16-Membered and 6-Membered TNF Peptides, Regulate TNFR1-Dependent Cytotoxic Activity of TNF
Source: Int J Mol Sci. 2024 Mar 31;25(7):3900. doi: 10.3390/ijms25073900 (PMC11011327; doi:10.3390/ijms25073900)
Supplement: Supplementary file 1 [file ijms-25-03900-s001.zip › ijms-2916028-supplementary.pdf]

Suplemental Matherials.

Supplemental Figure S1.

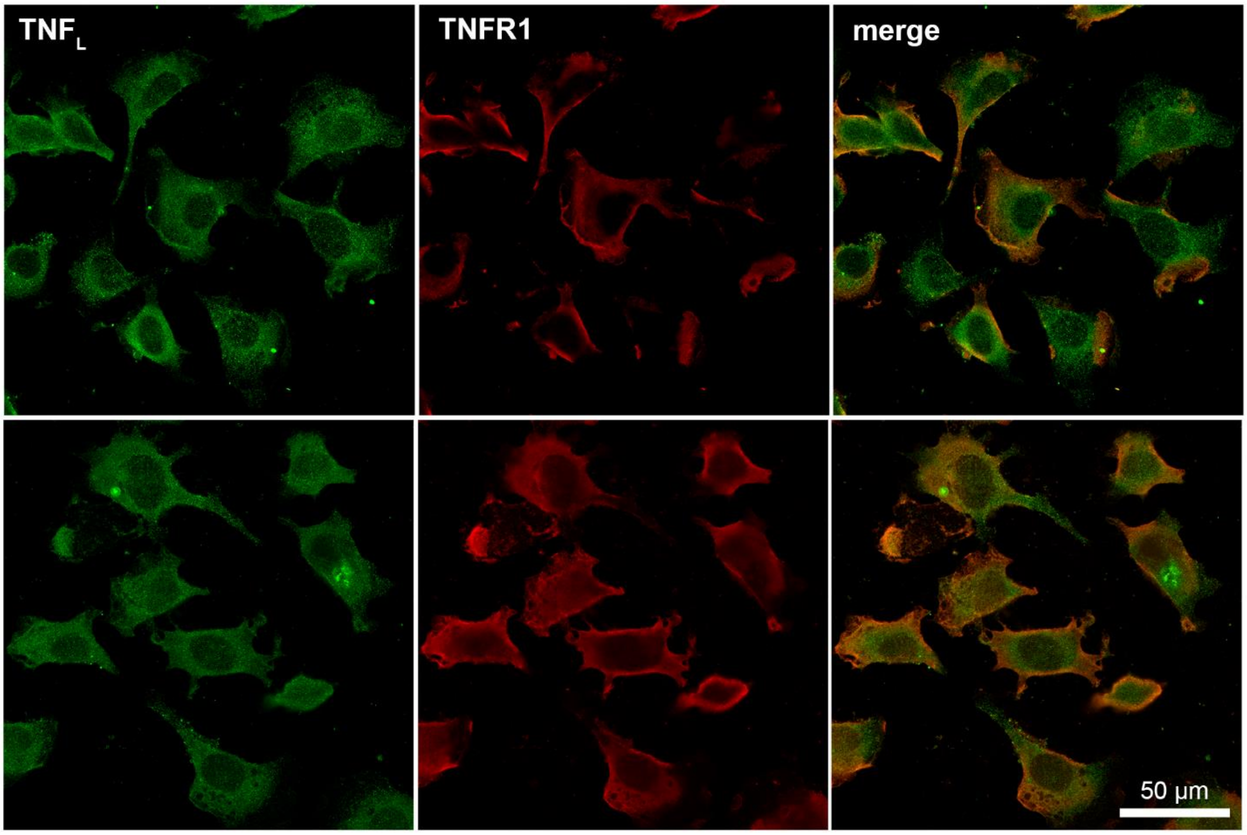

Typical confocal photos of TNF_L(green) and TNFR1(red) and layers superposition on the surface of L-929 cells.

Supplemental Figure S2.

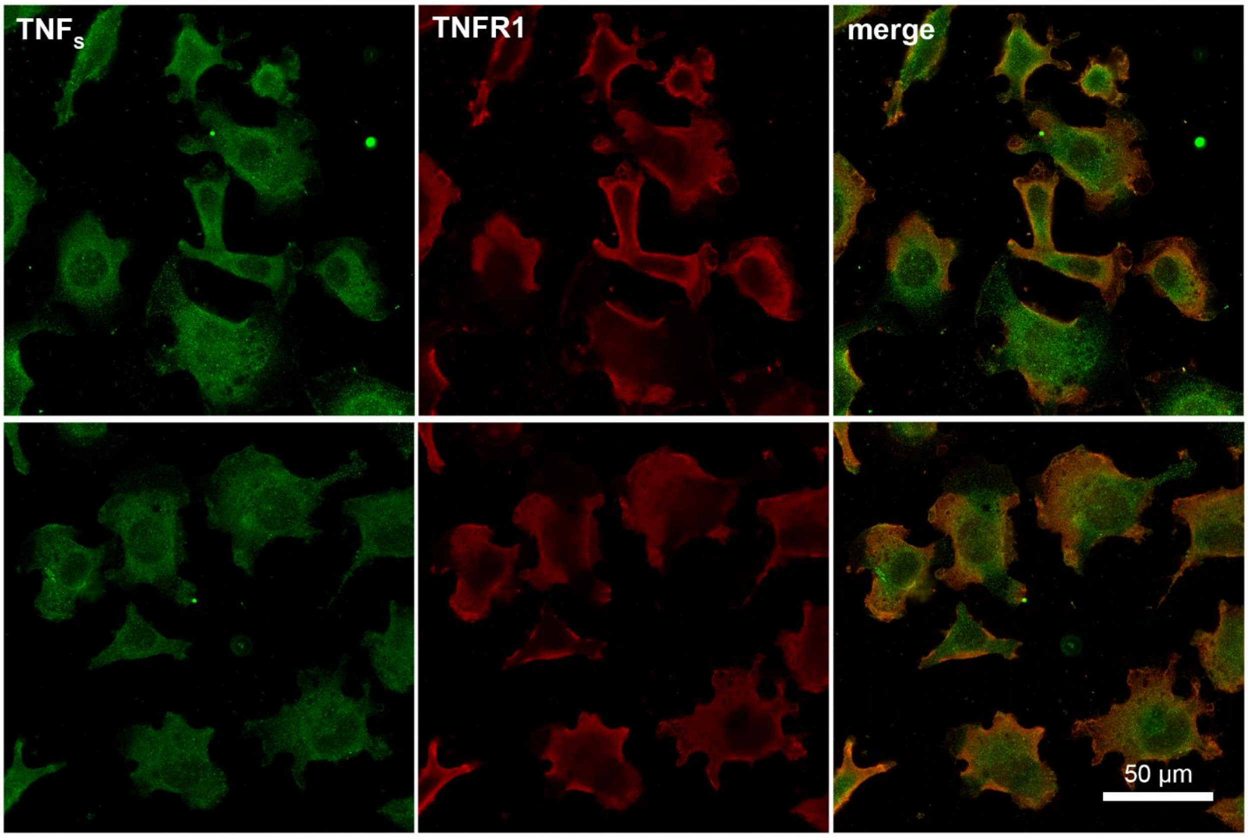

Typical confocal photos of TNFs(green) and TNFR1(red) and layers superposition on the surface of L929 cells.
